# Determination of Workload, Work Stress and Related Factors in Nursing Home Workers during the COVID-19 Pandemic in Turkey

**DOI:** 10.3390/ijerph20010160

**Published:** 2022-12-22

**Authors:** Şengül Akdeniz, Mustafa Çoban, Orhan Koç, Mehtap Pekesen, Nilufer Korkmaz Yaylagul, Seda Sönmez, Filiz Yetiz, Gülüşan Özgün Başıbüyük, Mehmet Efe, Ayşe Dericioğulları Ergun, Özlem Özgür, Melih Vural, Aslı Gözde Akış, İsmail Tufan

**Affiliations:** 1 Vocational School of Healthcare Services, Akdeniz University, 07070 Antalya, Turkey; 2 Department of Healthcare Management, Health Science Faculty, Akdeniz University, 07070 Antalya, Turkey; 3 Ministry of Family and Social Services, 06800 Ankara, Turkey; 4 Department of Gerontology, Health Science Faculty, Akdeniz University, 07058 Antalya, Turkey; 5 Faculty of Applied Science, Akdeniz University, 07070 Antalya, Turkey; 6 Department of Gerontology, Health Science Faculty, Mus Alparslan University, 49001 Muş, Turkey; 7 Vocational School of Healthcare Services, Mehmet Akif Ersoy University, 15030 Burdur, Turkey; 8 Private Neurology Clinic, 07070 Antalya, Turkey; 9 Doctorate Program, Institute of Health Science, Department of Gerontology, Akdeniz University, 07070 Antalya, Turkey

**Keywords:** COVID-19, pandemic, nursing home staff, workload, work stress

## Abstract

COVID-19 has caused a major crisis all over the world. To manage this crisis, a fixed shift system was applied to nursing home staff in Turkey to protect nursing home residents from the COVID-19 pandemic. Staff were not allowed to leave the institution during fixed shifts. It is thought that this practice for the COVID-19 outbreak, while protecting nursing home residents on the one hand, increased the workload and related stress of nursing home staff on the other hand. To the best of our knowledge, there is no study examining the workload and stress caused by the COVID-19 pandemic for nursing home staff in Turkey. The aim of this study was to examine the level of workload and work stress experienced by staff working in nursing homes during the COVID-19 pandemic in Turkey. Methods: A cross-sectional research design was used in the study. The sample of the study consisted of nursing home personnel working in nursing homes in the provinces of Istanbul, Ankara, Izmir and Antalya between October 2021 and January 2022. A personal information form and a workload and stress scale were used for collecting the data. Cluster analysis was performed with SPSS software. Results: In total, 154 nursing home personnel participated in the study. A statistically significant difference was found between the mean age of the two clusters. The first cluster was described as “old” and the second as “young”. Statistically significant and high values were found in the quantitative workload, qualitative workload, job organization, social work area and fatigue factors in the nursing home staff in the older participant cluster. Conclusion: The results of this study provide new information about the concepts of workload and work stress experienced during the COVID-19 pandemic in Turkey, which will serve as a guide for the management of future pandemics. Therefore, this study will contribute to the strategies to be followed in future pandemics in Turkey.

## 1. Introduction

The COVID-19 pandemic has been an experience that included a whole set of firsts for Turkey, as was the case in all countries around the world. COVID-19, which emerged at the end of December 2019 in Wuhan, China, was seen for the first time in Turkey on 11 March 2020. The first death occurred on 15 March 2020. As of 20 July 2022, the total number of patients infected with coronavirus in Turkey was 15.5 million, the number of recovered patients was 15.2 million and the number of patients who died was approximately 100,000 [1]. The social, economic, political, economic, administrative, legal, military, religious and cultural effects and consequences of the pandemic led to radical decisions. The Turkish government made many new arrangements as well as many support packages to minimize the economic effects of the pandemic [2]. The most memorable decision for the Turkish public, among the radical decisions taken to reduce the spread of the epidemic and maintain social distance, was imposing a lockdown for people aged 65 and over. Later, this was extended to people aged 20 and under [3].

Ten coronavirus guides were published by the Ministry of Family and Social Services, such as the “Information Guide for the Elderly” and the “Coronavirus Information Guide for the Elderly Over 65 Years of age with Chronic Disease”, during the process of the pandemic. In nursing homes, eye, ear and throat examinations and body temperature measurements were repeated every 6 h; hygiene equipment was supplied; a 7–10-day fixed shift system was established, which transitioned to a 14-day fixed-shift system on 7 April 2020; and nursing homes and elderly care and rehabilitation centers were closed to outside visits in an effort to prevent an increased number of deaths. During this period, the shift period was increased to 10 days and then 14 days in institutions providing elderly services, and personnel were prohibited from leaving the institution [4]. This may have helped to increase the workload and related stress of the COVID-19 pandemic experienced by the staff of these institutions, but empirical evidence of this has not been presented so far.

The nursing home sector in Turkey has a longer history than the care sector. Approximately 14,000 people are served in 163 nursing homes under the Ministry of Family and Social Affairs, with a capacity of 16,509. In addition, approximately 11,000 people are cared for in 267 private nursing homes, with a capacity of 17,508, and 2400 elderly people are cared for in 21 nursing homes in other public institutions, with a capacity of 3000. Currently, 27,113 elderly people live in 451 nursing homes that are either affiliated with the Ministry or private [5].

The population aged 65 and over in Turkey is 8.2 million [6]. The profession known as “elderly care technician” in Turkey is very new and is considered to be a type of patient care [7]. It has been ignored that policy, science and practitioners agree that elderly care is not just patient care [7,8]. The care profession requires a high degree of responsibility and a high standard of care. Primary caregivers who take care of the elderly experience more difficulties in physical, emotional, social, economic and work-related areas, since the individuals receiving care have both advanced age and disease. The findings of scientific studies on the care profession show that heavy pressure, which is generally perceived as stress, has negative consequences on the workers’ health. Tiring working conditions cause physical discomfort, decrease staff motivation and increase job dissatisfaction [7,9,10,11,12].

Stress is defined as varying degrees of exhaustion of individual performance due to workload. Workload causes negative experiences in the individual and is classified as stress if it has a repulsive nature. The workload should be adapted to the performance needs of the person so that the stress caused by the workload does not increase too much. Otherwise, the job will become stressful and become a threat with various consequences. All employees are affected by job stress due to the cognitive, emotional and behavioral demands arising from the content, organization and working environment of their jobs [13].

In the literature, many studies have been conducted in Turkey and other countries on the experience of workload and work stress of nursing home staff in the prepandemic period [9,10,11]. During the pandemic, many studies in the international literature focused on the workload and work stress experiences of nursing home staff [13,14,15,16,17,18,19]. In Turkey, since the beginning of the COVID-19 period, although there have been studies on the effects of COVID-19 on the health workers in health institutions, especially in hospitals [20,21,22,23], there has been no research on the workload and work stress of nursing home workers. The studies conducted in nursing homes in Turkey consist of studies on nursing home residents. There are also studies examining the field-specific effects of COVID-19, such as sports, tourism and financial indicators [24,25,26,27].

International studies conducted during the pandemic have shown that the spread of the COVID-19 pandemic around the world has caused psychological problems in healthcare workers such as anxiety about contagion, fear, anxiety about death, depression, general anxiety, insomnia and stress [13,18,28,29]. It has been determined that healthcare workers are exposed to more emotional strain than other professions because they experience the pain of difficult triage decisions and the pain of losing patients and colleagues [28]. An increase in the workload of healthcare workers and major changes in the routine work environment can cause emotional strain in employees [30]. It was determined that healthcare workers experienced increased workload, isolation and discrimination during the COVID-19 period, which caused them to experience physical fatigue, fear, emotional disturbances and sleep problems [19]. In a study conducted before the pandemic in Turkey, it was reported that the severity of working conditions in care services, the insufficient number of employees and structural deficiencies caused the employees to experience difficulties in their work and reduced the quality of the care services provided to the elderly in the elderly care process [9].

Considering that the workload and work stress levels of nursing home staff are important for the provision of quality care in nursing home care practice, the existing data on this issue are still incomplete, so more research is needed. Therefore, it is very important to reveal the impact of workload and stress on nursing home staff during the pandemic period in Turkey by addressing it in a holistic manner. Therefore, this study is important in terms of revealing the experiences of nursing home staff regarding the workload and work stress experienced following the process of increasing the mandatory shift period to 10 days and then to 14 days for the management of an extraordinary public health emergency in institutions providing elderly care in Turkey, alongside staff being prohibited from leaving the institution during this period.

The aim of this study was to determine the workload and stress levels of the working personnel and their determinants in nursing homes under the compulsory shift system during the COVID-19 pandemic in Turkey.

## 2. Materials and Methods

### 2.1. Participants

In total, 71 nursing homes in the provinces of Istanbul (24), Ankara (18), Izmir (16) and Antalya (13) were contacted, and detailed written information about the purpose and objectives of the survey was given to the management of 30 nursing homes that agreed to participate in the survey. All of the nursing homes included in the study serve under the Ministry of Family and Social Services. The nursing homes included in the study have a minimum capacity of 60 and a maximum capacity of 116, and these nursing homes accommodate elderly residents who require both normal care and special care. The nursing homes included in the study are located in the eastern, western and southern regions of the country, as well as in the four largest cities of the country. Twenty-three of the nursing homes included in the study are located in urban areas that are in a close relationship with the city, while seven of them are located in newly constructed areas that are further away from the city centers [31].

Written information about the research was given to 289 nursing home staff working in the 30 nursing homes that participated in the research. The consent of 154 people (participation rate = 53%) who agreed to answer our questions was obtained, and they were assured that their anonymity would be protected and that privacy would be strictly enforced. An agreement was made that a report of the survey’s results would be given to the nursing homes. This study was conducted in accordance with the principles of the Declaration of Helsinki. Ethical approval for the study was obtained from the National Society of Social and Applied Gerontology Ethics Committee, with the decision dated 20 November 2020 and numbered 176.14-86 Ethics-42/044.22.11.23.

### 2.2. Measurement

In this study, a personal information form, workload questionnaires and work stress questionnaires were used to collect the data [32]. In the survey, which was conducted for the first time in Turkey to examine the workload and stress of nursing home workers during the COVID-19 pandemic, two questionnaires designed by the Health Care and Welfare Care Professional Trade Association operating in Germany were used for institutions providing institutional care. At the stage when this research was planned, there was no measurement tool that was adapted to Turkish and for which the validity and reliability had been tested for assessing the individual perceptions of workload level of the employees of nursing homes and elderly care rehabilitation centers, so the scale used for researching German care institutions was translated into Turkish by two translators. The questionnaires were first translated into Turkish by the author of the study, who is a native German speaker who holds dual Turkish and German citizenship, and this translation was compared with the original questionnaire. By comparing the texts of the original German questionnaire and the translated questionnaire, the researchers and translators were able to agree on common statements. The Turkish translation of the questionnaire was evaluated by all authors of the study. Finally, the translated Turkish questionnaire was applied to a group working in a nursing home, which was not included in the sample of the study. According to the feedback obtained from the pilot questionnaire, minor word corrections were made in the final form of the questionnaire, and this was used as a data collection tool in the study. One of the questionnaires contained questions that detected workload and the other determined stress. Data were collected on quantitative and qualitative work stress, job organization, the social work environment, nonoccupational status and the physical and mental/mental status of nursing home workers through questions for which the scientific and practical importance has been proven. The only change we made to the questions is that a phrase such as “Compare with before the COVID-19 pandemic...” was always added to the beginning of the questions. Because of the lack of empirical data on prepandemic workload and work-related stress, this addition to the questions aimed to compare the current (pandemic) situation with the prepandemic status. The workload questionnaire consisted of 23 items. The questionnaire had five main sub-scales: quantitative workload, qualitative workload, work organization, social work environment and nonwork situation.

The 23-item workload questionnaire had 5 items in the quantitative workload subdimension, 5 items in the qualitative workload subdimension, 3 items in the job organization subdimension, 6 items in the social workplace subdimension and 4 items in the nonwork situation subdimension. The minimum and maximum scores obtained for the subdimensions were 0–30 for quantitative workload, 0–30 for qualitative workload, 0–12 for job organization, 0–24 for social workplace and 0–15 for nonwork situation.

The stress questionnaire consisted of 15 items. The scale had five main subsections: fatigue symptoms, job satisfaction, reactive protection, aversion to nursing home residents and emotional fatigue. The 15-item job stress questionnaire had 5 items in the fatigue subdimension, 3 items in the job satisfaction subdimension, 2 items in the reactive protection subdimension, 2 items in the subdimension of aversion toward nursing home residents and 3 items in the emotional fatigue subdimension. The minimum and maximum scores obtained for the subdimensions were 0–20 for fatigue, 0–8 for job satisfaction, 0–8 for reactive protection, 0–8 for aversion toward nursing home residents and 0–12 for emotional fatigue.

Both scales were evaluated on a 6-point Likert-type scale. Higher scores indicated that the perceptions of the work environment or workload were negative, and lower scores indicated that the workload was perceived positively. According to the findings of the study, statistically significant high values were found for the quantitative workload, qualitative workload, work organization, social workspace and fatigue factors of nursing home employees. Higher scores indicated that the perceptions of workload and stress in the work environment were highly negative.

### 2.3. Statistical Methods

The data were analyzed using Statistical Package for the Social Sciences (SPSS) Statistics Base Version 23, which has been licensed in Turkey. In the data analysis, the descriptive statistics were found first. Cluster analysis was then performed. Compared with basic statistical analysis, cluster analysis has the great advantage of accounting for more features simultaneously and allowing a comprehensive statement about the structure of the data. The fundamental analysis method, which is often very laborious, can be shortened considerably by this effective information gathering method. In many fields of application, the question of whether there is similarity between the objects of the study is important. The basis of this is the desire to bring similar objects together, that is, to classify them. Classification is the division of a set of objects into groups. The similarity matrix obtained from the data with the help of a proximity measure constitutes the starting point of the cluster algorithm, which aims to summarize the objects. In this context, a distinction between monothetic and polythetic algorithms can be made. The former uses only one variable in the grouping algorithms, but the advantage of clustering analysis lies in the simultaneous use of all descriptive properties in the process of grouping objects. In this survey, the hierarchical procedure was chosen. It started with the smallest cluster, so each object represented a cluster. The distance or similarity of all the objects included in the research was calculated. The two clusters (objects) that were closest were searched. The two objects (clusters) with the greatest similarity were merged into a new cluster. The distances between the new cluster and the remaining clusters were calculated. This process resulted in a reduced distance matrix. These steps were repeated until all the examined objects had been assigned to a cluster. In the questionnaire, the distance between objects was calculated with the Ward coefficient. Classification is the application of assignment rules to objects for which the cluster membership is unknown. It is carried out by considering the distance or similarity/dissimilarity between objects according to predefined properties [33,34]. In the survey, the workload and stress of nursing home personnel were classified according to selected characteristics and predefined cluster criteria. The nursing home personnel were presented with each question/statement, and the answers were classified in the process of cluster analysis. The subjectively perceived causes of an event can be classified according to two dimensions, namely, position (internal or external) relative to one’s own sphere of influence and variability over time (durable or variable). The SPSS software was used for cluster analysis. For significance, *p*-values less than 0.05 were accepted as significant.

In this research, solutions with 2, 3, 4, 5 and 6 clusters were designed through cluster analysis, and the differences between them were determined. The most logical seemed to be the 2-cluster solution. In this context, it was found that age was a determining factor, whereas gender was not a determining factor. A statistically significant difference was observed between the mean age of the two clusters formed. There were 52 people in the first cluster, and the average age of this group was 34.6 years (SD = 5.9). The average age of the 102 people in the second cluster was calculated to be 26.9 years (SD = 3.2). Here, the first cluster was described as “old” and the second as “young”. According to this finding, the age of the individual was effective in the answers given to the questions about workload and stress.

## 3. Results

### Descriptive Characteristics of the Study Participants

Due to cultural differences between the east and west of Turkey, nursing homes in Turkey are mostly established in the west of the country and in major cities. Therefore, this study was conducted on nursing home staff working in four large cities in the regions of the Aegean, Marmara, the Mediterranean and Central Anatolia in the west of the country. The study population consisted of 71 nursing homes in Istanbul (24), Ankara (18), Izmir (16) and Antalya (13). The sample of the study consisted of 154 nursing home staff working in 30 nursing homes (participation rate = 53%) who agreed to answer the survey questions. Of the 154 respondents, 45 were male (29.2%), and 109 were female (70.8%). Of these, 78 (50.6%) were married, and 76 (49.4%) were single. The mean age of the sample was calculated to be 29.5 (SD = 5.6). In total, 57 (37%) of the participants were nurses, 41 (27.6%) were elderly care technicians and 56 (36.4%) were certified assistant caregivers. According to gender, 77.8% of the men were elderly care technicians, and 22.2% were certified assistant caregivers. In contrast, 52.3% of the women were nurses, 5.5% were elderly care technicians and 42.2% were certified assistant caregivers. The first question posed to the participants was, “To what do you attribute the fact that you have successfully fulfilled your duties during the pandemic so far? Please tick one of the answer options”. Almost half of the participants (47.4%) decided on the answer of “professional ability”. The answer of “great effort” came in second place, with a rate of 27.3%. This was followed by those who answered, “It is difficult to describe”, with a rate of 16.9%. In last place were those who answered “coincidence”, with a rate of 8.4%. On the basis of these answers, the cluster analysis, the results of which are explained below, was performed.

According to Table 1, it was determined that the mean scores of workload and stress factors did not show a significant excessive deviation from the normal distribution. According to Table 1, statistically significant high values were found for quantitative workload, qualitative workload, work organization, social workspace and fatigue factors in nursing home staff. When evaluating the average values given in the Table 1, it should be remembered that the tendency to give a negative response increases as the scores for each scale become larger.

When evaluating the average values given in Table 2, it should be considered that the tendency to give a negative response increases as the scores for each scale become larger. For the older cluster of nursing home personnel, statistically significant higher values were found in five dimensions, namely in quantitative workload, qualitative workload, job organization, social work area and fatigue. In the other defined workload and stress dimensions, no difference was found between the young and old clusters; in other words, both of these clusters felt the same amount of workload and stress. For the Ward method to be used in cluster analysis, the measurement values are recommended to be compatible with the normal distribution with no correlation between the variables [33]. While the condition of a normal distribution was fulfilled, correlations were found for the variables of job organization, job satisfaction and aversion toward nursing home residents. When two clusters were created via cluster analysis by removing these three variables from the analysis, a slight change occurred, but there was no change in the general situation (the values given in parentheses in the table are the values calculated after removing the variables of job organization, job satisfaction and aversion toward nursing home residents).

**Figure 1:**
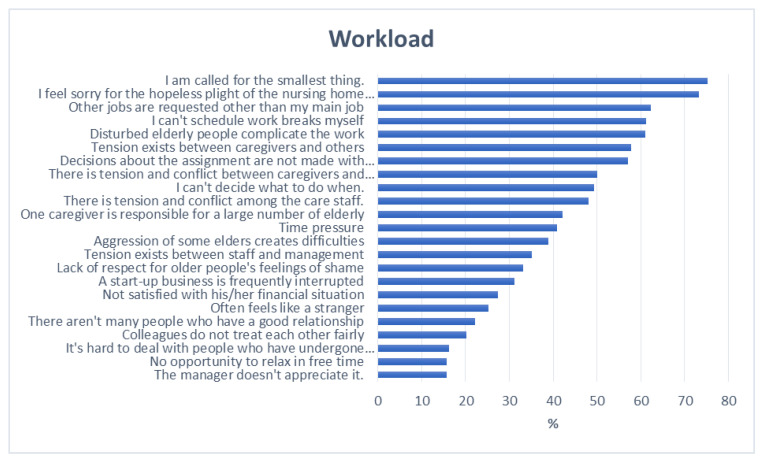
Participants’ answers regarding workload, as percentages (*n* = 154).

**Figure 2:**
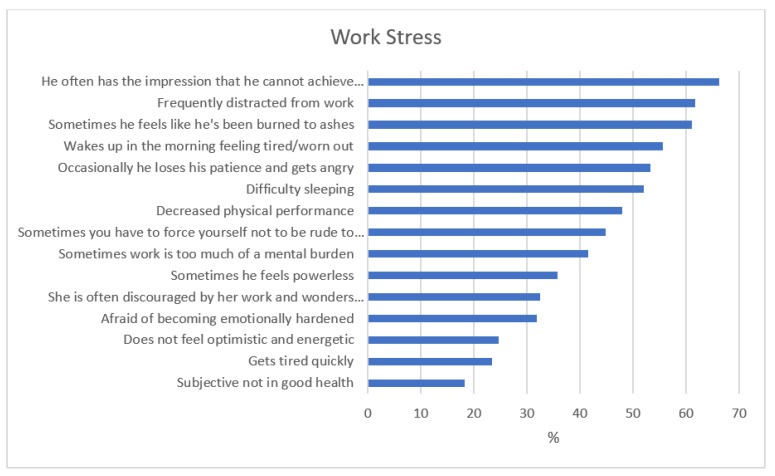
Participants’ answers about work stress, as percentages (*n* = 154).

On the basis of the answers given to the questions related to workload and work stress, it was found that the most important problems were related to job organization, the emotional pressure of the work and the relationships among the personnel. Figure 1 and Figure 2 show where the most and least problems occurred.

## 4. Discussion

This study provided an overview of an assessment of the workload and stress levels of nursing home staff working with the 14-day continuous shift system, which was implemented for the first time ever in nursing homes in Turkey during the pandemic period. The aim of this study was to investigate the workload and work stress levels experienced by the staff working in nursing homes during the COVID-19 pandemic in Turkey in the process of protecting both themselves and the nursing home residents. To the best of our knowledge, this is the first study to determine the workload and work stress following the strategy of increasing the required shift period to 10 days, then 14 days, and prohibiting the staff from leaving the institution during this period to manage the extraordinary public health emergency in institutions that provide elderly care in Turkey. This study revealed that the most important problems associated with workload and work stress in nursing home staff were related to work organization, the emotional pressure of the work and relationships among the staff. The innovative aspect of this study is that it contributes to the strategies to be followed by providing new information about the concepts that can serve as a guide in the management of future pandemics, especially the stress caused by the workload and work stress experienced during the COVID-19 pandemic in Turkey.

Turkey has carefully monitored and followed the outbreak from the very beginning. The Ministry of Health established the Coronavirus Scientific Committee. In line with the recommendations of the Scientific Committee chaired by the Minister of Health, many changes regarding the “new normal” were gradually implemented in social life. Among these measures, the Ministry of Family, Labor and Social Services instructed all institutions for the elderly and disabled providing inpatient services to work with 14-day fixed shifts. With the 14-day fixed shift system, all personnel (administrative, maintenance, cleaning and technical) working with the system were divided into two groups and each group stayed in the facility for 14 days per shift. Contact with social groups was cut off, and a PCR test had to be performed before starting work in the facility. The ability of the elderly and disabled individuals staying in institutions to leave was suspended, and visits to the institutions and social activities in the institutions were suspended for an indefinite period of time. The use of masks was mandatory in all inpatient institutions, and short briefings on prevention methods were given to the elderly and staff in line with the guidelines created by the scientific board on cleaning, hygiene and the COVID-19 pandemic [35,36].

While the fixed shift system in nursing homes and the closure of nursing homes to outside visits ensured the protection of nursing home residents, the prohibition of staff from leaving the institution during this period increased the workload related to the COVID-19 pandemic on the staff of these institutions and the related work stress. Although it is generally known that the staff working in institutions such as hospitals, nursing homes and nursing homes face a heavy workload, there was not enough information on workload and work-related stress among the many recommendations in the brochure published by the Ministry of Health, which included principles of prevention and control to reduce the risk of infection in nursing homes and elderly care centers within the context of the COVID-19 pandemic. We found that employees were affected by job stress caused by the cognitive, emotional and behavioral demands arising from the content, organization and working environment of the job. Alongside these assumptions, the age of the employees emerged as an important factor, according to the findings of this research, in which we measured the workload and work stress of nursing home staff in Turkey during the COVID-19 pandemic. When we compared the two groups, which we defined as “old” and “young” on the basis of the results of the cluster analysis, in terms of workload and stress, we determined that those in the older cluster had higher levels of workload and work stress.

The COVID-19 pandemic crisis has unexpectedly exacerbated the challenges of working conditions for nursing home staff [37]. The workload and work stress of nursing home staff working in a high-stress environment increased because of the uncertainties regarding COVID-19, the long and tiring working hours, and disruptions in the provision of protective equipment. There is no doubt that the COVID-19 pandemic increased the workload and work stress of the staff working in nursing homes through the constantly increasing incidence and mortality rates. However, the fact that the workload and work stress of the older staff in the study were higher than those of the younger staff may be attributed to their internal efforts and high work performance in attempts to cope with the current crisis resulting from their longer work experience and professional participation compared with the younger staff.

In the study, it was determined that nursing home staff serving the elderly population, which was defined as disadvantaged because these people were at high risk during the COVID-19 pandemic, had difficulty in responding to the increasing demands and experienced emotional fatigue. This result is consistent with other studies attempting to associate work stress, workload and perceived health during the COVID-19 pandemic [37,38,39,40]. These results reveal findings that indicate the risk of deterioration in the physical and/or mental health of nursing home staff during the COVID-19 pandemic. In addition to all these, the concerns of nursing home staff about catching the disease themselves and transmitting it to the family members they are in contact with suggest the risk of experiencing emotional exhaustion and even compassion fatigue.

Zhang et al. (2018), in their meta-analysis conducted before the pandemic, concluded that age, gender and length of service were not significantly associated with the prevalence rates of compassion fatigue or burnout [41]. Nevertheless, the negative and challenging working conditions of the pandemic have been a game changer. Compassion, a psychological state of well-being that includes people’s cognitive and affective functions, is a vital motivational factor that manifests itself in the effort to meet the needs of others through the caregiving process [42]. However, although helping others during the disruptive COVID-19 pandemic increased the satisfaction of compassion, it is possible that older staff working in an environment characterized by a continuous series of stressful events experienced compassion fatigue and emotional burnout [43,44]. This is because of the series of stressful situations in which caregivers were unable to meet their own emotional needs, and this occupational stress is directly related to the mismatch between job demands and availability, which can lead to compassion fatigue and eventually burnout [45].

This study revealed that the most important problems associated with workload and work stress in nursing home staff were related to work organization, the emotional pressure of the work and relationships among the staff. The fact that the workload and work stress of the older personnel were higher than those of the younger personnel suggests that they had more work experience and that this situation was likely to bring about burnout and compassion fatigue. Considering the workload-related variables one by one, it was seen that workload can arise from both the demands of nursing home residents and the status of nursing home residents. It was also found that the workload originating from job organization was also high. This may be one of the reasons for the tension between the staff and nursing home managers. One of the factors that aggravated workload is tension among the nursing home workers (care staff) and between the care staff and others outside the care staff, which indicated that “team spirit” was running low. In addition, the increase in workload due to time constraints seemed to be quite high.

Workplaces are environments in which people face the most stress. Believing that one cannot cope with a job, feeling uncomfortable about a job and burnout syndrome are among the issues most contributing to work-related stress among nursing home personnel. Studies have shown that individuals with good self-control experience less stress [46,47]. An ideal level of self-control leads to an increase in work performance, success, self-esteem, interpersonal relationships and positive emotions. However, constantly striving to maintain such an ideal level of self-control can be exhausting. As revealed in this study, the energy used for self-control decreases after each new self-control action, and when repetitive actions that require self-control prevent the recovery of limited energy, the individual’s energy is depleted, leading to stress. Moreover, when self-control demands have to be met simultaneously or successively, the energy expended by the individual is greater than the sum of the energy expended for each action separately; thus, the individual experiences higher levels of stress. According to the results obtained from this study, the quarantine measures further increased the psycho-social risks to which nursing home workers were already exposed. In addition, during the pandemic, there have been qualitative and quantitative changes in the workloads of nursing home staff [46,47,48,49]. The periods of quarantine and social isolation affected both the staff and residents of nursing homes [50]. In addition, the frequent sharing of news with various communication tools during the pandemic and the fact that the contents of this shared news mostly reported frightening numbers of cases and deaths increased the fear of the pandemic and increased work stress [51]. Social support, appropriate breaks, experience sharing and on-the-job training, especially from managers, colleagues, and family members, create positive conditions that help to combat work stress. These interventions aim to improve employees’ awareness, knowledge, skills and coping skills for stress management. In summary, practices such as stress management training, cognitive behavioral therapy, anger control, relaxation exercises and meditation are among the stress-reducing interventions used for nursing home workers [12,21,48,49].

### Study Strengths and Weaknesses

The greatest strength of the study is that it involved comprehensive research that included data evaluated in multiple nursing homes. Another strength of the study is that it evaluated the workload and stress levels experienced by the staff during the COVID-19 pandemic in Turkey, when restrictions on issues such as taking leave, resignation and working hours were imposed on healthcare professionals, and the elderly were always cared for by the same staff in an uninterrupted 14-day shift system imposed on nursing homes and elderly care centers during a period when the uncertainties of the pandemic were intense. The study also had some limitations. First, in Turkey, nursing homes are mostly established in the west of the country and in large cities. This study was conducted only on nursing home staff working in the four largest cities of the regions of the Aegean, Marmara, the Mediterranean and Central Anatolia in the west of the country. Nursing home staff in the eastern regions of Turkey could not be included in the sample. The second limitation was that data could not be obtained by observation because of the pandemic.

## 5. Conclusions

This study revealed that the most important problems associated with workload and work stress in nursing home staff were related to work organization, the emotional pressure of the work and relationships among the staff. When we compared the two groups, which we defined as “old” and “young” on the basis of the results of the cluster analysis, in terms of workload and stress, it was determined that those in the older cluster experienced higher levels of workload and work stress. This study provides an overview of the assessment of the workload and stress levels of nursing home staff working under the 14-day continuous shift system, which was implemented for the first time ever in nursing homes in Turkey during the pandemic period. In addition, it is thought that these findings will make important contributions to nursing home staff in terms of planning and implementing the services to be provided in this direction by defining the factors associated with workload and work stress in the future.

It is important both socially and scientifically to reveal the current situation of nursing home employees, whose workload has increased with the pandemic and whose working conditions have become more difficult and who have faced various leave and dismissal bans. It can be accepted that the need for nursing and eventide homes will increase in Turkey in the coming years because of the aging population. The construction of the necessary facilities to meet this need will not create a large problem. The main problems will arise from the working opportunities of the nursing and eventide home staff, the professional competence of that staff and the job organization. Care insurance will be the most important incentive factor for the development of these sectors in Turkey. Care insurance will create new possibilities. Both nursing and eventide homes will want to take advantage of these opportunities. Care insurance alone will not be sufficient. It is also necessary to make changes in the training of personnel so they can achieve professional competence. In addition to medical care, personnel equipped with social, gerontological, gerontopsychological and gerontopsychiatric knowledge will be needed.

## Figures and Tables

**Table 1:** Workload and stress factors determined from the variables and normal distribution compatibility tests.

	Mean Score	(SD)	Minimum	Maximum	Kolmogorov–Smirnov Z	Asymptotic Significance
Quantitative workload (5 items)	18.10	3.95	0	30	0.980	0.29
Qualitative workload (5 items)	17.58	3.23	0	30	0.964	0.31
Job organization (3 items)	7.83	2.21	0	12	1.257	0.09
Social workplace (6 items)	16.21	2.59	0	24	1.181	0.12
Nonwork situation (4 items)	9.012	2.44	0	15	1.263	0.08
Fatigue (5 items)	11.47	3.20	0	20	1.294	0.07
Job satisfaction (3 items)	4.04	1.92	0	8	1.422	0.04 *
Reactive protection (2 items)	4.38	2.03	0	8	1.307	0.07
Aversion toward nursing home residents (2 items)	4.83	1.91	0	8	1.321	0.06
Emotional fatigue (3 items)	8.18	2.34	0	12	1.247	0.09

* The normal distribution criterion was not met for the variable of job satisfaction.

**Table 2:** Average values of the dimensions of workload and stress for the different clusters.

	Mean Value of the Sample N = 154	Cluster			
		1 (“Old”) N = 52 Mean Value	2 (“Young”) N = 102 Mean Value	1 (“Old”) N = 52 SD	2 (“Young”) N = 102 SD
Quantitative workload	18.1	21.0 (21.9)	16.6 (16.3)	3.8	3.1
Qualitative workload	17.6	20.2 (20.1)	16.2 (16.4)	2.7	2.6
Job organization	7.8	9.4	7.0	1.8	1.9
Social workspace	16.2	17.8 (17.1)	15.0 (15.3)	2.6	2.2
Nonwork situation	9.0	9.3 (10.1)	8.9 (8.5)	2.3	2.5
Fatigue	11.5	14.3 (13.9)	10.1 (10.3)	2.5	2.5
Job satisfaction	4.0	4.2	3.9	2.3	1.7
Reactive protection	4.4	4.5 (48)	4.3 (4.2)	2.4	1.8
Aversion toward nursing home residents	4.8	4.7	4.9	2.2	1.8
Emotional fatigue	8.2	8.8 (8.6)	7.8 (8.0)	2.0	2.4

## Data Availability

The data presented in this study are available on request from the corresponding author. The data are not publicly available due to the agreement with participating organizations.
